# Impact of collateral status on functional outcomes in acute cardioembolic stroke after successful endovascular thrombectomy

**DOI:** 10.3389/fneur.2026.1782442

**Published:** 2026-03-31

**Authors:** Jun Chen, Fanghui Li, Mingjun Pu, Zhonglun Chen, Zhaokun Li, Xinyu Zou, Jiacai Zuo, Ying Cao, Ling Yang, Min Luo, Yufeng Tang, Rui Zeng

**Affiliations:** 1 Department of Cardiology, West China Hospital, Sichuan University, Chengdu, Sichuan, China; 2 Mianyang Central Hospital, School of Medicine, University of Electronic Science and Technology of China, Mianyang, China; 3 Department of Neurology, Mianyang Third People's Hospital, Mianyang, China

**Keywords:** cardioembolic stroke, collateral status, endovascular thrombectomy, functional outcome, large vessel occlusion

## Abstract

**Background:**

Collateral circulation sustains the ischemic penumbra during acute large vessel occlusion (LVO). However, its prognostic significance in cardioembolic stroke (CS) patients following successful endovascular thrombectomy (EVT) remains unclear. We investigated whether collateral status influences functional outcomes in acute CS patients achieving successful recanalization [modified Thrombolysis in Cerebral Infarction (mTICI) grade ≥2b].

**Methods:**

We retrospectively analyzed consecutive CS patients achieving successful recanalization with EVT. Collateral status was assessed using the American Society of Interventional and Therapeutic Neuroradiology/Society of Interventional Radiology (ASITN/SIR) grading system on digital subtraction angiography (DSA). The primary outcome was functional independence [modified Rankin Scale (mRS) 0–2] at 3 months. Secondary outcomes included excellent outcome (mRS 0–1) at 3 months, in-hospital and 3-month mortality, and PH (parenchymal hematoma type 1c or 2) within 72 h. Multivariable logistic regression and subgroup analyses were performed to identify independent associations.

**Results:**

Among 354 patients, 211 (59.6%) had good collaterals (ASITN/SIR 2–4) and 143 (40.4%) had poor collaterals (ASITN/SIR 0–1). After adjustment for confounders, good collateral status independently predicted functional independence [adjusted odds ratio (aOR), 2.07; 95% CI, 1.13–3.83; *p* = 0.020], with rates of 61.6% versus 37.1% in the good versus poor collateral groups. Good collaterals were also independently associated with excellent outcome (aOR, 1.89; 95% CI, 1.03–3.50; *p* = 0.042), lower in-hospital mortality (aOR, 0.24; 95% CI, 0.08–0.63; *p* = 0.006), lower 3-month mortality (aOR, 0.39; 95% CI, 0.18–0.78; *p* = 0.009), and reduced PH rates within 72 h (aOR, 0.51; 95% CI, 0.27–0.93; *p* = 0.029). The association between collateral status and functional independence was modified by baseline Alberta Stroke Program Early CT Score (ASPECTS) (*P* for interaction = 0.007).

**Conclusion:**

In CS patients achieving successful recanalization, good collateral status is independently associated with improved functional independence and excellent outcome, reduced PH rates within 72 h, and lower in-hospital mortality and 3-month mortality. Collateral assessment may enhance prognostic stratification, particularly in patients with lower baseline ASPECTS.

## Introduction

Endovascular thrombectomy (EVT) has become the standard treatment for acute ischemic stroke due to large vessel occlusion (LVO), with multiple randomized controlled trials demonstrating substantial benefits in improving functional outcomes (1, 2). Despite achieving successful recanalization, however, approximately half of patients fail to achieve functional independence at 90 days (3). Cardioembolic stroke (CS) represents the most common etiology of LVO, accounting for 50% of cases, and is characterized by distinct pathophysiological features including larger thrombus burden, specific patterns of tissue injury and worse pre-EVT collateral status compared to LVO strokes caused by large artery atherosclerosis (LAA) (4, 5).

Collateral circulation sustains the ischemic penumbra during arterial occlusion, and good collaterals have been associated with better outcomes after EVT (6, 7). However, emerging evidence suggests that the prognostic impact of collaterals may differ by stroke etiology, with potentially distinct effects in cardioembolic versus LAA stroke (8). Unlike LAA, where chronic hypoperfusion allows gradual collateral recruitment, CS is characterized by abrupt vessel occlusion without preceding collateral development (5). This pathophysiological difference may influence how collaterals modulate treatment response and outcomes.

Despite being the predominant etiology requiring EVT, studies examining collateral status as a prognostic factor specifically in CS patients achieving successful recanalization remain scarce. Furthermore, it remains unclear whether clinical and imaging characteristics—such as baseline infarct burden, stroke severity, and patient demographics—modify the prognostic impact of collateral status in this population. We therefore investigated whether collateral status independently influences functional outcomes, hemorrhagic complications, and mortality in CS patients achieving successful recanalization with EVT. We hypothesized that better pre-intervention collateral status would be associated with improved 3-month functional outcomes and explored potential effect modifiers of this relationship through subgroup analyses.

## Methods

### Study design and patient population

This retrospective cohort study included consecutive patients with acute ischemic stroke due to cardioembolism who underwent EVT for LVO between January 2018 and December 2024 at Mianyang Central Hospital, a comprehensive stroke center. The study was approved by the biomedical ethics committee of Mianyang Central Hospital (Approval No. S20220310-01), and written informed consent was obtained from all participants or their legally authorized representatives. Eligible patients were ≥18 years with radiologically confirmed occlusion involving the intracranial internal carotid artery (ICA) and/or the M1 or M2 segments of the middle cerebral artery (MCA). Exclusion criteria were: (1) onset-to- puncture time (OTP) > 24 h; (2) stroke etiology other than cardioembolism; (3) inability to assess collateral circulation on digital subtraction angiography (DSA); (4) baseline Alberta Stroke Program Early CT Score (ASPECTS) < 3; (5) prior mRS > 1; (6) final modified Thrombolysis in Cerebral Infarction (mTICI) grade <2b; and (7) lost to follow-up at 3 months.

### Data collection

Demographic data, vascular risk factors, medical history, laboratory parameters, treatment details, and EVT procedural information including time metrics were prospectively collected. Baseline stroke severity was assessed using the National Institutes of Health Stroke Scale (NIHSS) on admission. Premorbid functional status was determined by mRS. Stroke etiology was classified according to the Trial of Org 10,172 in Acute Stroke Treatment (TOAST) criteria by an experienced neurologist, based on comprehensive assessment of medical history, clinical presentation, and imaging findings (9). The diagnosis of cardioembolism required objective evidence from documented patient history, admission electrocardiogram, or inpatient cardiac monitoring. The primary etiology of cardioembolic stroke was determined by experienced neurologists based on established literature and expert consensus (10, 11).

### Imaging protocol and collateral assessment

Baseline ASPECTS was assessed on non-contrast computed Tomography (CT) by an experienced neuroradiologist. Collateral status was assessed on DSA images using the ASITN/SIR by an experienced neuroradiologist (12). The ASITN/SIR grading system ranges from 0 to 4: Grade 0 = no collateral vessels visible to the ischemic site; Grade 1 = slow collaterals to the periphery with persistence of defect; Grade 2 = rapid collaterals to the periphery with persistence of some defect and only partial ischemic territory filling; Grade 3 = slow but complete collateral flow of the entire ischemic bed by the late venous phase; Grade 4 = complete and rapid collateral flow to the entire ischemic territory by retrograde perfusion. For analysis, ASITN/SIR scores of 0–1 were classified as poor collateral status and scores of 2–4 as good collateral status (12). Successful recanalization was defined as a final mTICI score of 2b or 3 (13).

### Endpoint and follow-up

The primary outcome was functional independence, defined as mRS 0–2 at 3 months after successful EVT. Secondary outcomes included: (1) excellent functional outcome (mRS 0–1) at 3 months; (2) in-hospital mortality; and (3) 3-month mortality. The safety outcome was the occurrence of parenchymal hematoma (PH), defined as Heidelberg Bleeding Classification type 1c (PH1) or class 2 (PH2), within 72 h of EVT, assessed on follow-up CT or magnetic resonance imaging (14). Functional outcomes at 3 months were assessed by certified stroke nurses blinded to baseline information via structured telephone interviews or in-person clinic visits.

### Statistical analysis

Continuous variables were expressed as mean ± standard deviation for normally distributed data or median [interquartile range (IQR)] for non-normally distributed data, and compared using Student’s t-test or Mann–Whitney U test, respectively. Categorical variables were presented as numbers (percentages) and compared using chi-square test or Fisher’s exact test as appropriate.

Multivariable logistic regression analyses were performed to identify independent associations of collateral status with clinical outcomes, including functional independence, excellent functional outcome, in-hospital mortality, 3-month mortality, and PH within 72 h. Variables with *p* < 0.05 in univariable analyses were entered into the multivariable models. Multiple imputation with chained equations was used to handle missing data for variables included in adjusted models. Results were reported as odds ratios (ORs) with 95% confidence intervals (CI). Exploratory subgroup analyses were conducted to assess potential effect modification by prespecified variables, including age (≤ 75 vs. > 75 years), baseline NIHSS (≤ 15 vs. > 15), baseline ASPECTS (< 8 vs. ≥ 8), puncture-to-recanalization time (PTR) (≤ 60 vs. > 60 min), diabetes mellitus, and final mTICI grade (2b vs. 3). Interactions were tested using multiplicative interaction terms in adjusted logistic regression models.

All statistical tests were two-tailed, and *p* < 0.05 was considered statistically significant. Statistical analyses were performed using R software version 4.0 (R Foundation for Statistical Computing, Vienna, Austria).

## Results

### Baseline characteristics

**Figure 1:**
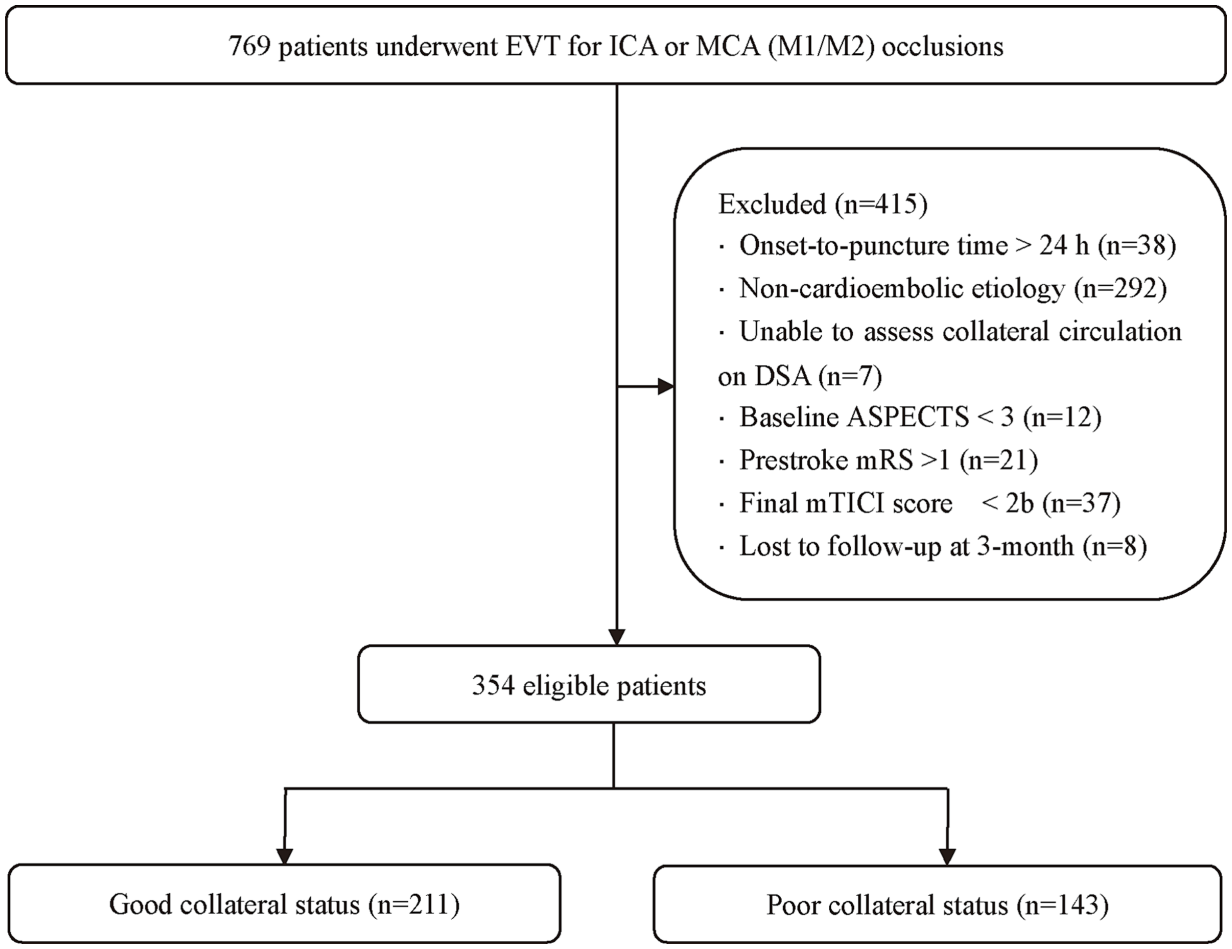
Flowchart of patient inclusion and exclusion. EVT, endovascular thrombectomy; ICA, internal carotid artery; MCA, middle cerebral artery; ASPECTS, Alberta Stroke Program Early CT Score; mTICI, modified Thrombolysis in Cerebral Infarction.

Among 769 consecutive patients who underwent EVT for ICA or MCA (M1/M2) occlusions during the study period, 415 were excluded: OTP > 24 h (*n* = 38), non-cardioembolic stroke etiology (*n* = 292), inability to assess collateral circulation on DSA (*n* = 7), baseline ASPECTS < 3 (*n* = 12), prior mRS > 1 (*n* = 21), final mTICI < 2b (*n* = 37), and loss to follow-up at 3 months (*n* = 8). The final cohort comprised 354 patients, of whom 211 (59.6%) had good collaterals and 143 (40.4%) had poor collaterals (Figure 1).

The median age was 75 years (IQR, 67–81), 143 (40.4%) were male, the mean baseline NIHSS score was 15.0 ± 5.8, and the median baseline ASPECTS was 9 (IQR, 7–10). Intravenous thrombolysis was administered prior to EVT in 103 patients (29.1%). Atrial fibrillation was the predominant etiology of cardioembolic stroke (*n* = 297, 83.9%), valvular heart disease (*n* = 28, 7.9%), prosthetic heart valve (*n* = 13, 3.7%), atrial flutter (*n* = 4, 1.1%), hypertrophic obstructive cardiomyopathy (*n* = 4, 1.1%), acute coronary syndrome (*n* = 2, 0.6%), dilated cardiomyopathy (*n* = 2, 0.6%), patent foramen ovale (n = 2, 0.6%), heart failure (*n* = 1, 0.3%), and infective endocarditis (*n* = 1, 0.3%). Baseline characteristics stratified by collateral status are presented in Table 1.

**Table 1:** Baseline characteristics of patients stratified by collateral status.

Characteristics	Good collateral (*n* = 211)	Poor collateral (*n* = 143)	*p*-value
Age, y, median (IQR)	75 (67–80)	75 (68–82)	0.624
Male, *n* (%)	82 (38.9)	61 (42.7)	0.475
Transfer, *n* (%)	140 (66.4)	90 (62.9)	0.509
Stroke history, *n* (%)	34 (16.1)	21 (14.7)	0.716
Wake-up stroke, *n* (%)	52 (24.6)	43 (30.1)	0.258
Systolic blood pressure, mmHg, median (IQR)	150 (135.5–170)	142 (129–166)	0.038
Diastolic blood pressure, mmHg, median (IQR)	86 (76–99)	84 (71.5–96)	0.238
Diabetes mellitus, *n* (%)	42 (19.9)	36 (25.2)	0.240
Hypertension, *n* (%)	101 (47.9)	72 (50.3)	0.647
Hypercholesterolemia, *n* (%)	12 (5.7)	8 (5.6)	0.970
Antiplatelet use, *n* (%)	20 (9.5)	14 (9.8)	0.922
Anticoagulation use, *n* (%)	37 (17.5)	24 (16.8)	0.854
Statin use, *n* (%)	30 (14.2)	19 (13.3)	0.803
Prior mRS, *n* (%)			0.566
0	202 (95.7)	135 (94.4)	
1	9 (4.3)	8 (5.6)	
Glucose on admission, mmol/L, median (IQR)	7.5 (6.3–8.8)	7.3 (6.4–9.1)	0.737
INR, median (IQR)	1.1 (1–1.1)	1.1 (1–1.1)	0.260
BNP, pg./mL, median (IQR)	1,500 (774.1–3057.5)	1,590 (961.6–2,974)	0.552
Admission NIHSS, mean ± SD	13.8 (5.7)	16.7 (5.6)	< 0.001
Baseline ASPECTS, median (IQR)	9 (7,10)	8 (6,10)	< 0.001
Intravenous thrombolysis, *n* (%)	68 (32.2)	35 (24.5)	0.115
First-pass effect, *n* (%)	96 (45.5)	43 (30.1)	0.004
First procedural characteristics, *n* (%)			0.740
Stent retriever first	75 (35.5)	46 (32.2)	
Aspiration first	135 (64)	97 (67.8)	
Angioplasty first	1 (0.5)	0 (0)	
Number of retrieval attempts, median (IQR)	2 (1–3)	2 (1–3)	0.001
Site of occlusion, *n* (%)			< 0.001
ICA	33 (15.6)	68 (47.6)	
M1 segment of MCA	124 (58.8)	72 (50.3)	
M2 segment of MCA	54 (25.6)	3 (2.1)	
OTP, min, median (IQR)	320 (225–595)	319 (200–552.5)	0.333
PTR, min, median (IQR)	62 (40–90)	56 (40.5–87.5)	0.456
OTR, min, median (IQR)	395 (292–653.5)	374 (274.5–616.5)	0.335
mTICI, *n* (%)			0.588
2b	108 (51.2)	69 (48.3)	
3	103 (48.8)	74 (51.7)	
The primary etiology of cardioembolic stroke, *n* (%)			0.881
AF	176 (83.4)	121 (84.6)	
Heart failure	0 (0)	1 (0.7)	
Acute coronary syndrome	2 (0.9)	0 (0)	
Patent foramen ovale	1 (0.5)	1 (0.7)	
Infective endocarditis	1 (0.5)	0 (0)	
Valvular heart disease	15 (7.1)	13 (9.1)	
Prosthetic heart valve	8 (3.8)	5 (3.5)	
Dilated cardiomyopathy	2 (0.9)	0 (0)	
Atrial flutter	3 (1.4)	1 (0.7)	
Hypertrophic obstructive cardiomyopathy	3 (1.4)	1 (0.7)	
PH within 72 h, *n* (%)	24 (11.4)	32 (22.4)	0.005
mRS at 3 months, *n* (%)			< 0.001
0	43 (20.4)	13 (9.1)	
1	64 (30.3)	27 (18.9)	
2	23 (10.9)	13 (9.1)	
3	22 (10.4)	10 (7)	
4	19 (9)	13 (9.1)	
5	20 (9.5)	25 (17.5)	
6	20 (9.5)	42 (29.4)	
mRS 0–1 at 3 months, *n* (%)	107 (50.7)	40 (28)	< 0.001
mRS 0–2 at 3 months, *n* (%)	130 (61.6)	53 (37.1)	< 0.001
Morality in hospital, *n* (%)	6 (2.8)	18 (12.6)	< 0.001
Morality within 3 months, *n* (%)	20 (9.5)	42 (29.4)	< 0.001

IQR, interquartile range; mRS, modified Rankin Scale score; INR, international normalized ratio; BNP, B-type natriuretic peptide; NIHSS, National Institutes of Health Stroke Scale; ASPECTS, Alberta Stroke Program Early CT Score; ICA, internal carotid artery; MCA, middle cerebral artery; OTP, onset-to-puncture time; PTR, puncture-to-recanalization time; OTR, onset-to-recanalization time.; mTICI, modified Thrombolysis in Cerebral Infarction; AF, atrial fibrillation; PH, parenchymal hematoma.

### Collateral status

Compared with patients with poor collaterals, those with good collaterals had lower baseline NIHSS scores (13.8 ± 5.7 vs. 16.7 ± 5.6, *p* < 0.001), higher baseline ASPECTS [median, 9 (IQR, 7–10) vs. 8 (IQR, 6–10); *p* < 0.001], and higher rates of first pass effect (45.5% vs. 30.1%, *p* = 0.004). Patients with good collaterals also had different occlusion site distributions: lower rates of ICA occlusion (15.6% vs. 47.6%), and higher rates of M1 (58.8% vs. 50.3%) and M2 occlusions (25.6% vs. 2.1%). The primary etiology of cardioembolic stroke did not differ significantly between the two groups (*p* = 0.881).

### Association between collateral status and clinical outcomes

**Figure 2:**
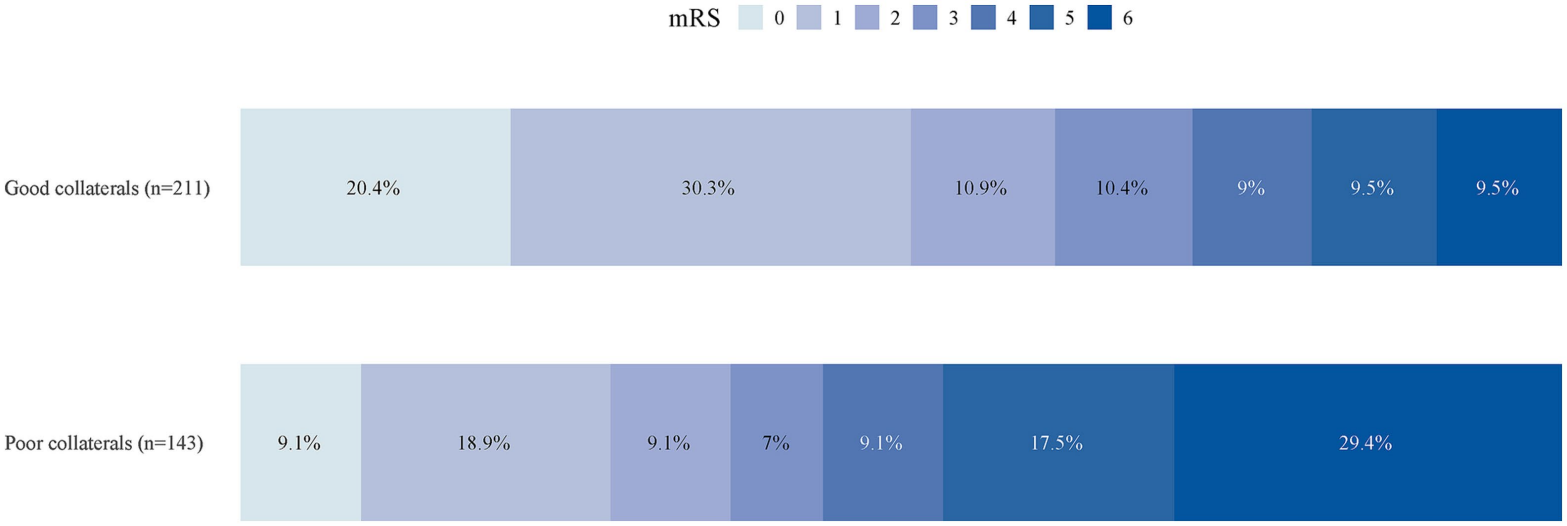
The distribution of 3-month mRS scores based on collateral status. mRS, modified Rankin Scale score.

In univariable analyses, patients with good collaterals had significantly higher rates of functional independence compared with those with poor collaterals (61.6% vs. 37.1%, *p* < 0.001), and excellent functional outcome (50.7% vs. 28.0%, *p* < 0.001) (Table 1). The distribution of 3-month mRS scores varied significantly based on collateral status (Figure 2).

After adjustemt for including age, transfer, stroke history, systolic blood pressure, admission glucose, admission NIHSS score, admission ASPECTS, first-pass effect, number of retrieval attempts, site of occlusion, final mTICI grade, PTR, PH within 72 h, diabetes mellitus, hypertension, and B-type natriuretic peptide (BNP), good collateral status remained independently associated with functional independence (adjusted OR, 2.07; 95% CI, 1.13–3.83; *p* = 0.020) (Table 2). Similarly, after multivariable adjustment for age, stroke history, systolic blood pressure, admission glucose, admission NIHSS score, admission ASPECTS, first-pass effect, number of retrieval attempts, site of occlusion, PTR, PH within 72 h, diabetes mellitus, and final mTICI grade, good collaterals independently predicted excellent functional outcome (aOR, 1.89; 95% CI, 1.03–3.50; *p* = 0.042).

**Table 2:** Multivariable logistic regression analysis of primary and secondary outcomes based on collateral status.

Outcomes	Good collateral	Poor collateral	Univariate analysis		Multivariate analysis	
			OR 95%CI	*p*-value	OR 95%CI	*p*-value
Primary outcome						
Functional independence^a^	130 (61.6)	53 (37.1)	2.73 (1.76, 4.23)	<0.001	2.07 (1.13, 3.83)	0.020
Secondary outcomes						
Excellent outcome^b^	107 (50.7)	40 (28)	2.65 (1.68,4.17)	<0.001	1.89 (1.03, 3.50)	0.042
PH within 72 hours^c^	24 (11.4)	32 (22.4)	0.45 (0.25, 0.079)	0.006	0.51 (0.27, 0.93)	0.029
In-hospital mortality^d^	6 (2.8)	18 (12.6)	0.20 (0.8, 0.53)	0.001	0.24 (0.08, 0.63)	0.006
3-month mortality^e^	20 (9.5)	42 (29.4)	0.25 (0.14, 0.45)	< 0.001	0.39 (0.18, 0.78)	0.009

a Adjusted for age, transfer, stroke history, systolic blood pressure, glucose on admission, admission NIHSS score, admission ASPECTS, first pass effect, number of retrieval attempts, site of occlusion, final mTICI grade, PTR, PH within 72 h, diabetes mellitus, hypertension and BNP.

b Adjusted for age, stroke history, systolic blood pressure, glucose on admission, admission NIHSS score, admission ASPECTS, first pass effect, number of retrieval attempts, site of occlusion, PTR, PH within 72 h, diabetes mellitus and final mTICI grade.

c Adjusted for transfer, antiplatelet use, baseline ASPECTS, intravenous thrombolysis, OTR and INR.

d Adjusted for wake-up stroke, glucose on admission, admission NIHSS score, OTR and diabetes mellitus.

e Adjusted for transfer, glucose on admission, admission NIHSS score, admission ASPECTS, number of retrieval attempts, site of occlusion, diabetes mellitus, INR, BNP, PH within 72 h.

OR, odds ratio; CI, confidence interval; PH, parenchymal hematoma; NIHSS, National Institutes of Health Stroke Scale; ASPECTS, Alberta Stroke Program Early CT Score; mTICI, modified Thrombolysis in Cerebral Infarction; PTR, puncture to recanalization time; PH, parenchymal hematoma; BNP, B-type natriuretic peptide; OTR, onset to recanalization time; INR, international normalized ratio.

PH within 72 h occurred less frequently in patients with good collaterals than in those with poor collaterals (11.4% vs. 22.4%; *p* = 0.005). After adjustment for transfer, antiplatelet use, admission ASPECTS, intravenous thrombolysis, onset to recanalization (OTR) time and international normalized ratio (INR), good collateral status was independently associated with lower risk of PH within 72 h (aOR, 0.51; 95% CI, 0.27–0.93; *p* = 0.029).

In-hospital mortality was significantly lower in patients with good collaterals (2.8% vs. 12.6%; *p* < 0.001), as was 3-month mortality (9.5% vs. 29.4%; *p* < 0.001). In multivariable analysis adjusted for wake-up stroke, admission glucose, admission NIHSS score, OTR and diabetes mellitus, good collateral status independently predicted lower in-hospital mortality (aOR, 0.24; 95% CI, 0.08–0.63; *p* = 0.006). Similarly, after adjustment for transfer, admission glucose, admission NIHSS score, admission ASPECTS, number of retrieval attempts, site of occlusion, diabetes mellitus, INR, BNP, and PH within 72 h, good collaterals remained independently associated with lower 3-month mortality (aOR, 0.39; 95% CI, 0.18–0.78; *p* = 0.009).

### Subgroup analyses

**Figure 3:**
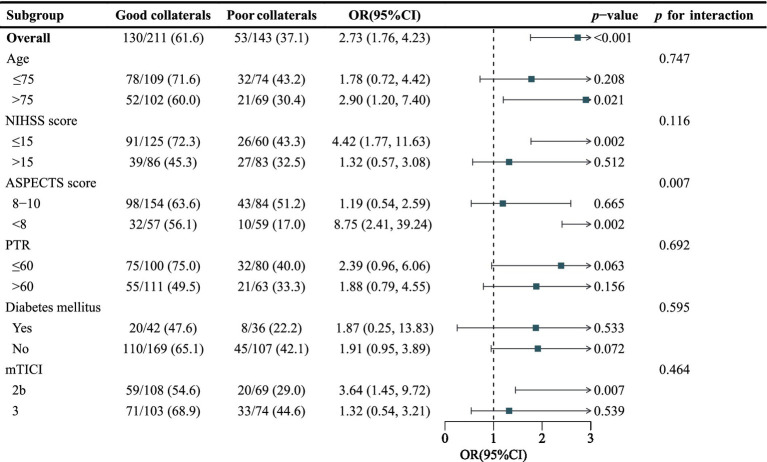
Collateral status and functional independence: Subgroup analysis interaction in the overall cohort. Subgroup analyses revealed that the association between collateral status and functional independence was modified by baseline ASPECTS (*p* for interaction = 0.007); NIHSS, National Institutes of Health Stroke Scale; ASPECTS, Alberta Stroke Program Early CT Score; PTR, puncture to recanalization time; AF, atrial fibrillation; mTICI, modified Thrombolysis in Cerebral Infarction.

Subgroup analyses revealed that the association between collateral status and functional independence was modified by admission ASPECTS (*p* for interaction = 0.007) (Figure 3). The benefit of good collateral status was substantially greater in patients with ASPECTS < 8 (OR, 8.75; 95% CI, 2.41–39.24) than in those with ASPECTS of 8–10 (OR, 1.19; 95% CI, 0.54–2.59). No significant interaction effects were observed for the other prespecified variables, including age, admission NIHSS score, PTR time, diabetes mellitus, or final mTICI grade (all *p* for interaction > 0.05).

## Discussion

In this retrospective cohort study of patients with acute CS who underwent successful EVT, we found that good collateral status was independently associated with significantly better functional outcomes at 3 months, reduced PH with 72 h, and lower mortality. Importantly, we identified a significant interaction between collateral status and baseline ASPECTS, with the beneficial effect of good collaterals being particularly pronounced in patients with ASPECTS <8.

Our findings demonstrate that collateral status provides robust prognostic information across multiple clinically relevant outcomes in CS patients achieving successful recanalization. Good collaterals were associated not only with functional independence (aOR, 2.07), but also with reduced PH within 72 h (aOR, 0.51), lower in-hospital mortality (aOR, 0.24), and lower 3-month mortality (aOR, 0.39). These findings are consistent with previous studies demonstrating the prognostic importance of collateral status in acute ischemic stroke (15, 16). However, prior studies have largely included heterogeneous stroke etiologies, and data specifically evaluating the role of collaterals in patients with CS achieving successful recanalization are limited.

Prior studies demonstrated that whole-brain arterial collateral status based on CTA or DSA for acute anterior circulation LVO were associated with improved functional outcomes in patients with successful vessel recanalization following reperfusion therapy (17–19). However, these studies lacked stratified analysis by stroke etiology in EVT patients. Another study demonstrated that in cardioembolism patients, good collaterals were associated with a higher chance of good functional outcome, lower mRS at 3 months, but were not associated with lower risk of symptomatic intracranial hemorrhage (8). However, this study did not analyze mortality outcomes, nor did it perform subgroup analysis of collateral status. In contrast, our study specifically enrolled CS with successful recanalization and utilized the gold standard DSA to evaluate collateral status. Beyond 3-month mRS assessment, we incorporated endpoints including PH with 72 h and all-cause mortality both in-hospital and at 3 months into our analysis.

The pathophysiology of CS differs fundamentally from LAA-related stroke. In patients with LAA, the chronic hypoperfusion prior to the index stroke allows more time for collateral vessel recruitment. And in cardioembolic stroke, the abrupt onset fails to provide sufficient time for collateral formation, leading to rapid cerebral ischemia and hypoxia that can cause swift expansion of both the infarct core and penumbra within a short period. Previous studies have found that good collateral status is associated with reduced infarct expansion and slower stroke progression, suggesting that collateral circulation plays an important role in limiting the expansion of the infarct core (19, 20). The mechanisms underlying the association between poor collaterals and adverse outcomes after successful recanalization are multifactorial. Experimental evidence from animal models demonstrates that insufficient leptomeningeal collaterals fail to sustain perfusion to distal arterial segments during ischemia, resulting in their collapse and a consequent loss of cerebrovascular autoregulation, which manifests as impaired vasoreactivity to hypercapnic challenge (21). Upon recanalization, these functionally impaired vessels are unable to appropriately regulate reperfusion, resulting in a rapid and uncontrolled hyperemic response characterized by perfusion exceeding baseline levels and accompanied by a cascade of pathological events these include mitochondrial dysfunction with reactive oxygen species generation, capillary and myocyte swelling, microvascular leukocyte adhesion with no-reflow phenomenon, and increased capillary permeability (21–23). Collectively, these processes ultimately contribute to hemorrhagic transformation and unfavorable functional outcomes (21–23).

Our exploratory subgroup analysis revealed a significant interaction between collateral status and ASPECTS (*p* = 0.007), providing important insights into the factors modifying the prognostic value of collateral status following successful recanalization in CS. Specifically, the protective effect of good collaterals on functional independence was substantially amplified in patients with ASPECTS < 8 (aOR, 8.75; 95% CI, 2.41–39.24) compared with those with ASPECTS ≥ 8 (aOR, 1.19; 95% CI, 0.54–2.59). No significant interactions were detected between collateral status and age, baseline NIHSS score, PTR, diabetes mellitus or recanalization grade (all *P* for interaction > 0.05); nevertheless, point estimates consistently favored good collaterals across all subgroups. The MR CLEAN-LATE trial demonstrated that good collaterals were essential for benefit in patients with anterior circulation LVO in the extended 6- to 24-h time window (24). Emerging evidence suggests that collateral assessment combined with ASPECTS evaluation enhances prognostic stratification, as good collaterals correlate with higher ASPECTS scores, slower infarct progression, and smaller final infarct volumes (18, 20, 25). Mechanistically, good collaterals sustain oxygen and glucose delivery to the penumbral tissue, preventing the progression from reversible oligemia to irreversible infarction and maintaining cellular energy metabolism until reperfusion is achieved (20, 26). The significant interaction observed between collateral status and ASPECTS suggests that the benefit of good collaterals may be particularly relevant in patients with lower ASPECTS scores, who have more extensive ischemic burden but also greater potential for tissue salvage with adequate collateral support. These findings may inform future patient selection strategies and enhance prognostic accuracy in CS patients undergoing EVT.

This study has several limitations. First, the retrospective, single-center design may limit the generalizability of our findings to other populations and healthcare settings, and the potential for selection bias cannot be entirely excluded. Second, patients with baseline ASPECTS less than 3 were excluded, which may have enriched our cohort with patients having better baseline conditions and potentially limited the applicability of our findings to patients with very large infarct cores. Third, the study period spanned 7 years, during which EVT techniques, devices, and perioperative management strategies evolved, potentially introducing temporal confounding. Fourth, despite multivariable adjustment, residual confounding from unmeasured variables such as infarct location heterogeneity, and anticoagulation status cannot be ruled out. Fifth, the relatively modest sample size may have limited statistical power for detecting interactions in some subgroup analyses, particularly in smaller subgroups. Sixth, we did not assess other imaging biomarkers of tissue viability, such as CT perfusion parameters or ischemic core volumes, which might provide complementary prognostic information alongside collateral status and ASPECTS. Finally, collateral circulation was assessed on DSA rather than on baseline CTA. While DSA remains the gold standard for collateral evaluation, it is performed after the decision for EVT has been made. The applicability of our findings to CTA-based collateral assessment requires further validation.

## Conclusion

In conclusion, good collateral status independently associated with improved functional independence and excellent outcome, reduced PH within 72 h, and lower mortality in CS patients achieving successful recanalization with EVT. The prognostic value of collateral assessment is particularly pronounced in patients with lower ASPECTS. These findings suggest that collateral status assessment may enhance prognostic stratification and inform clinical decision-making in CS patients undergoing EVT. Prospective multicenter validation studies and investigation of collateral-guided patient selection and management strategies represent important directions for future research.

## Data Availability

Data are available from the corresponding author upon reasonable request.
